# Proto-oncogene FAM83A contributes to casein kinase 1–mediated mitochondrial maintenance and white adipocyte differentiation

**DOI:** 10.1016/j.jbc.2022.102339

**Published:** 2022-08-02

**Authors:** Kuilong Huang, Zhihao Jia, Haoran Li, Ying Peng, Xiaochang Chen, Nanjian Luo, Tongxing Song, Yingqian Wang, Xin’e Shi, Shihuan Kuang, Gongshe Yang

**Affiliations:** 1 College of Animal Science and Technology, Northwest A&F University, Yangling, Shaanxi, China; 2 Department of Animal Sciences, Purdue University, West Lafayette, Indiana, USA; 3 Cambridge-Suda Genomic Resource Center, Soochow University, Suzhou, China; 4 Shaanxi Key Laboratory of Ischemic Cardiovascular Disease, Institute of Basic and Translational Medicine, Xi'an, Shaanxi, China

**Keywords:** FAM83A, adipose, mitochondria, energy metabolism, CK1, AT, adipose tissue, DEG, differential expression gene, eWAT, epididymal white AT, FBS, fetal bovine serum, GTT, glucose tolerance test, HFD, high-fat diet, ITT, insulin tolerance test, iWAT, inguinal white AT, MPTP, mitochondrial permeability transition pore, NCD, normal chow diet, RT-qPCR, reverse transcription quantitative PCR, sgRNA, single guide RNA, WAT, white AT

## Abstract

Family with sequence similarity 83 A (FAM83A) is a newly discovered proto-oncogene that has been shown to play key roles in various cancers. However, the function of FAM83A in other physiological processes is not well known. Here, we report a novel function of FAM83A in adipocyte differentiation. We used an adipocyte-targeting fusion oligopeptide (FITC-ATS-9R) to deliver a FAM83A-sgRNA/Cas9 plasmid to knockdown *Fam83a* (ATS/sg-FAM83A) in white adipose tissue in mice, which resulted in reduced white adipose tissue mass, smaller adipocytes, and mitochondrial damage that was aggravated by a high-fat diet. In cultured 3T3-L1 adipocytes, we found loss or knockdown of *Fam83a* significantly repressed lipid droplet formation and downregulated the expression of lipogenic genes and proteins. Furthermore, inhibition of *Fam83a* decreased mitochondrial ATP production through blockage of the electron transport chain, associated with enhanced apoptosis. Mechanistically, we demonstrate FAM83A interacts with casein kinase 1 (CK1) and promotes the permeability of the mitochondrial outer membrane. Furthermore, loss of *Fam83a* in adipocytes hampered the formation of the TOM40 complex and impeded CK1-driven lipogenesis. Taken together, these results establish FAM83A as a critical regulator of mitochondria maintenance during adipogenesis.

Adipose tissue (AT) serves as an important energy storage organ and plays key roles in maintaining the metabolic health. Inadequate AT (lipodystrophy) results in an insufficient energy reservation in response to environmental change, while excessive accumulation of AT could lead to obesity (1). Obesity is becoming a global epidemic in recent decade, and obesity-associated diseases, in particular, type II diabetes has posed great threats to human health. As dysfunctional AT is usually correlated with abnormal systemic energy metabolism, homeostasis of AT is essential to prevent metabolic diseases (2).

Mitochondria are the main supplier of energy for various types of cells (3, 4). Adipocyte differentiation is a very energy-intensive process (5), which relies on normal mitochondria function. Mitochondria are filamentous and organized in a continuous meshwork in adipogenic precursors. In contrast, lipid-laden mature adipocytes are dispersed around lipid drops (6). Mitochondria are a highly dynamic organelle that could rapidly reorganize to promptly meet metabolic demands. Mitochondrial fusion and fission directly affect lipid accumulation in AT (7), and the structural integrity of mitochondrial membranes in AT is closely linked to organismal aging (8). Moreover, it has been reported that knockdown of mitochondrial transcription factor in AT leads to mitochondrial damage and inhibits lipid droplet formation (9). Taken together, mitochondria provide essential energy to adipocytes and thus play an important role in the differentiation of adipocytes.

The casein kinase 1 (CK1) family possesses serine/threonine kinase activity and are involved in the regulation of cell differentiation, proliferation, chromosome segregation, and circadian rhythms in multiple cell types through interacting with different proteins (10, 11). Specifically, CK1 plays a critical and evolutionarily conserved role in the outer membrane of mitochondria. CK1 phosphorylates TOM22 (translocase of outer mitochondrial membrane 22) at Thr57 and stimulates the assembly of TOM22 and TOM20, which facilitates the assembly of TOM40 complex (12, 13). The TOM40 complex is important for maintaining the stability of mitochondrial outer membrane protein channels (14, 15). In addition, CK1 has been proved to be a positive regulator of adipogenesis (16, 17). Therefore, whether CK1 promotes adipogenesis through stabilizing mitochondria outer membrane protein is worth to be investigated.

The FAM83 family proteins contain a DUF1669 domain and a phospholipase D-like motif domain at their N terminus but lack the catalytic activity of phospholipase D (18). *Fam83a* is an important oncogene that widely and highly expressed in various cancers, especially in non–small cell lung cancer, hepatocellular carcinoma, and breast cancer (19, 20, 21). In RNA-seq data where KO of *Zfp217* led to a disrupted adipogenic differentiation of 3T3-L1 preadipocyte, the expression level of *Fam83a* is significantly upregulated (22), indicating that FAM83A may plays an inhibitory role in adipogenesis.

Here, by inhibiting *Fam83a*, we show a previously undescribed function of FAM83A in regulating mitochondria maintenance during adipogenesis of white adipocytes. Moreover, ATS/sg-FAM83A mice resist diet-induced obesity while loss or knockdown of FAM83A inhibits 3T3-L1 adipocyte differentiation. Mechanistically, FAM83A binds to CK1, which promotes the assembly of the TOM40 complex that maintains normal mitochondrial function in adipocytes.

## Results

### FAM83A is highly related to adipocyte differentiation and is partially located in the mitochondria

**Figure 1:**
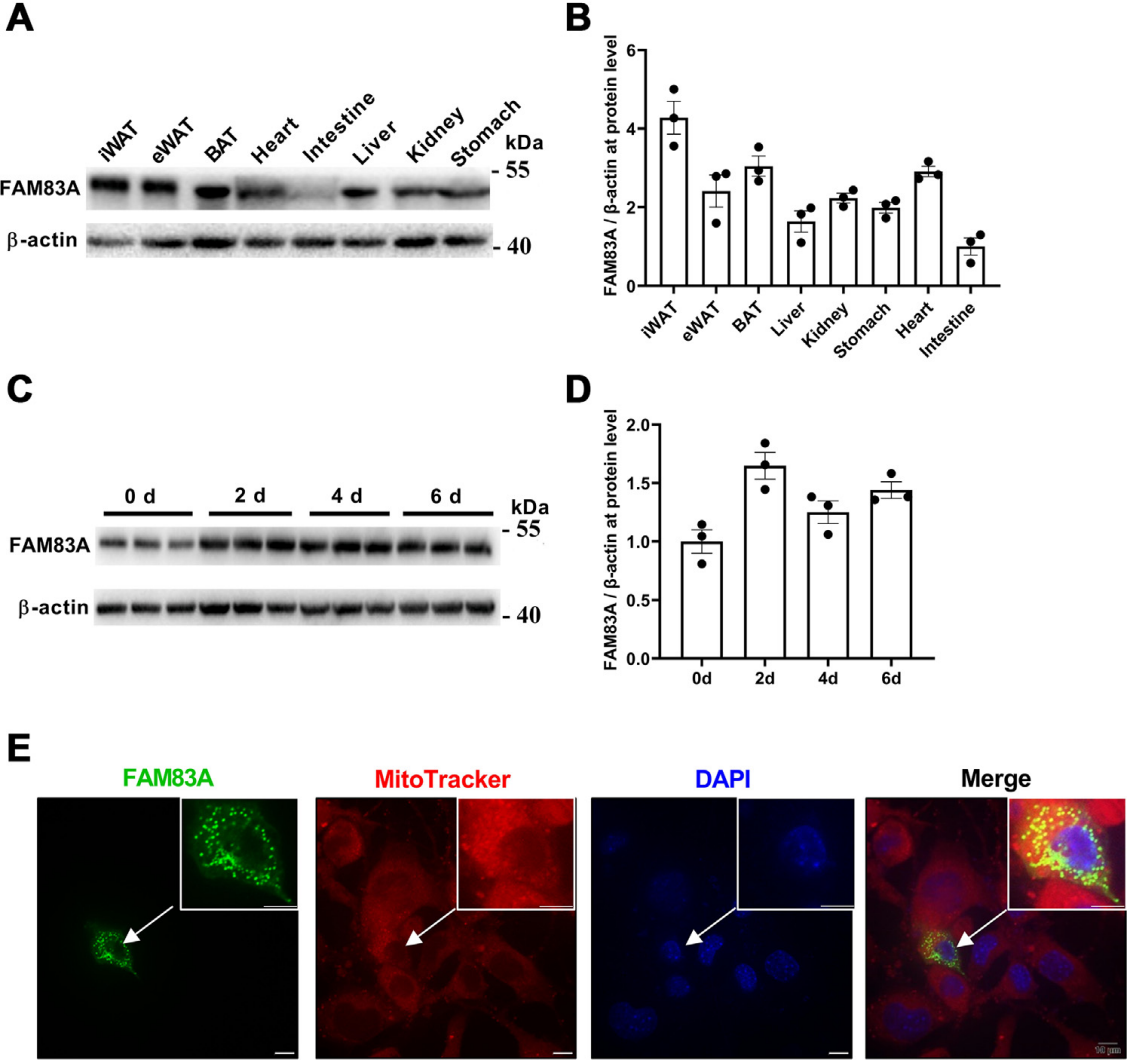
**The spatiotemporal expression pattern of *Fam83a* in mice and times and its subcellular distribution.***A* and *B*, FAM83A proteins were detected in multiple tissues of mice by Western blot (n ≥ 3). *C* and *D*, FAM83A proteins expression during the adipogenic differentiation in 3T3-L1 cell line were detected using Western blot (n = 4). *E*, subcellular distribution of FAM83A with the help of confocal laser. The scale bar represents 10 μm. Data are represented as means ± SEM. Significance was determined using *t* tests. ∗*p* < 0.05, ∗∗*p* < 0.01.

Firstly, we surveyed the expression pattern of Fam83a in relation to adipogenic differentiation. Western blot results showed that *Fam83a* was widely expressed in various tissues in mice, with relatively higher expression levels in ATs, especially in epididymal white AT (eWAT) and inguinal white AT (iWAT) (Fig. 1, *A* and *B*). During adipogenic differentiation of 3T3-L1 preadipocytes, the expression level of *Fam83a* increased upon adipogenic stimuli (Fig. 1, *C* and *D*). We further overexpressed the FAM83A-EGFP fusion protein in 3T3-L1 cells with a vector (FAM83A-N-EGFP) to detect the subcellular localization of FAM83A. Interestingly, FAM83A (indicated by EGFP signal) was mainly localized in the cytoplasm of 3T3-L1 cells, partially colocalized with mitochondria (indicated by mito-Tracker) (Fig. 1*E*). The results indicated a potential involvement of FAM83A in adipogenic differentiation, possibly in a mitochondria-related manner.

### White adipose knockdown of *Fam83a* mediated by FITC-ATS-9R and FAM83A-sgRNA/Cas9 oligoplexes reduces eWAT mass in mice on normal chow diet

**Figure 2:**
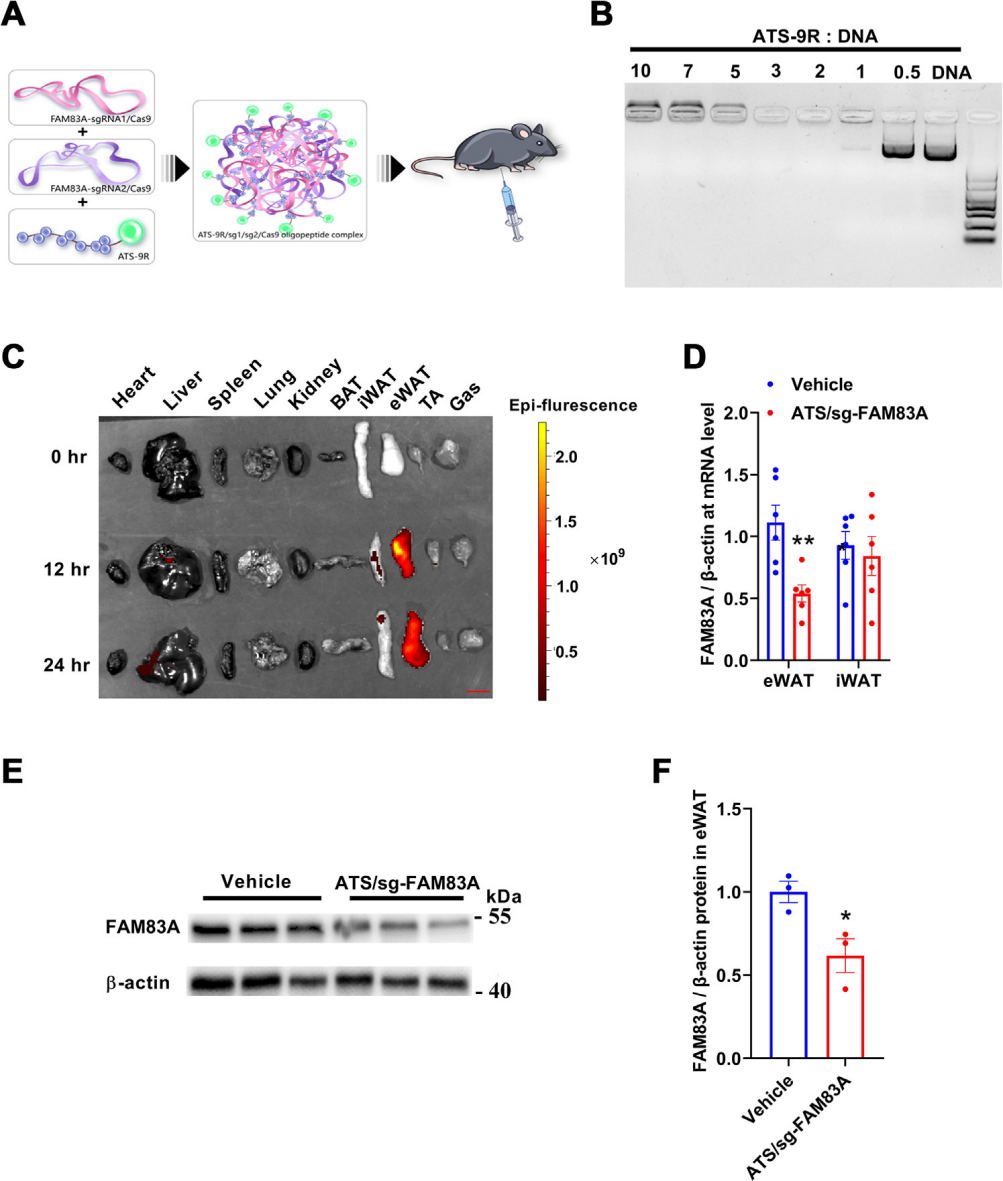
**Generation of an adipose-specific *Fam83a* KO mouse model.***A*, diagram of the procedure to generate adipose-specific *Fam8a* KO (ATS/sg-FAM83A) mice. *B*, the agarose gel electrophoresis image showing the binding efficiency of FITC-ATS-9R to FAM83A-sgRNA/Cas9 DNA. From right to left: DNA: FITC-ATS-9R = 1:0.5, 1:1, 1:2, 1:3, 1:5, 1:7, 1:10. *C*, FITC fluorescence to trace FITC-ATS-9R peptide in mice fed with normal chow diet (NCD). The scale bar represents 1 cm. *D*, the KO efficiency of ATS/sg-FAM83A complex in eWAT and iWAT of NCD mice was tested at mRNA levels with RT-qPCR (n = 6). *E* and *F*, the KO efficiency of ATS/sg-FAM83A complex in eWAT of NCD mice was tested with Western blot (n = 3). eWAT, epididymal white adipose tissue; iWAT, inguinal white adipose tissue; RT-qPCR, reverse transcription quantitative PCR.

According to a previous research (23), FITC-ATS-9R could specifically deliver plasmids into white AT (WAT). Therefore, we generated a FITC-ATS-9R and FAM83A–single guide RNA (sgRNA)/Cas9 complex to induce adipose-specific *Fam83a* knockdown (ATS/sg-FAM83A) in mice (Fig. 2*A*). The binding efficiency of FITC-ATS-9R with FAM83A-sgRNA/Cas9 was tested using DNA electrophoresis, and a ratio of FITC-ATS-9R to FAM83A-sgRNA/Cas9 over 2:1 was determined to get an acceptable results (Fig. 2*B*), and a final ratio of 3:1 was chosen in following study according to a previous report (24). After 12 and 24 h of i.p. injection, the fluorescence of FITC-ATS-9R was found mainly within the eWAT, with a small amount in the iWAT and liver, but not in other major organs or tissues (Fig. 2*C*). Consistently, the mRNA expression of *Fam83a* was significantly reduced in eWAT but not iWAT (Fig. 2*D*). In addition, FAM83A protein level was significantly decreased in eWAT of ATS/sg-FAM83A mice (Fig. 2, *E* and *F*). The results aforementioned that *Fam83a* could be effectively knocked down *via* FITC-ATS-9R-mediated target delivery of sgRNA/Cas9.

### ATS/sg-FAM83A mice are resistant to high-fat diet–induced obesity

**Figure 3:**
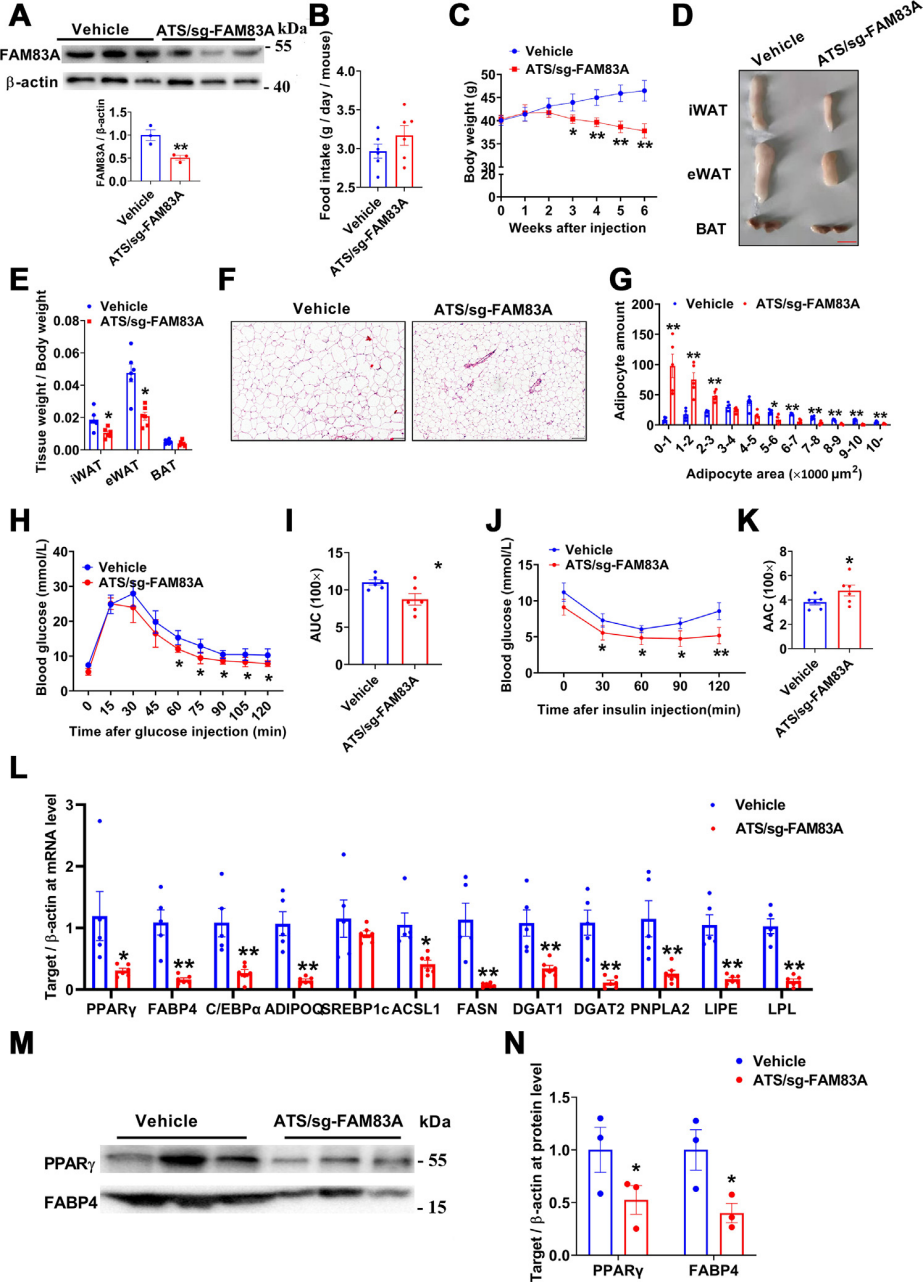
**KO of *Fam83a* in WAT resisted HFD-induced obesity.***A*, the KO efficiency in eWAT was determined at protein level with Western blot. *B*, food intake during the experiment (n = 6). *C*, growth curve of HFD mice (n = 6). *D*, morphological comparison of iWAT, eWAT, and BAT (n = 6). The scale bar represents 1 cm. *E*, tissue weights of iWAT, eWAT, and BAT. *F*, H&E staining of eWAT section. *G*, size of adipocytes in eWAT (n = 6). The scale bar represents 100 μm. *H*–*K*, GTT and ITT tests (n = 6). *L*, changes of adipogenesis-, lipogenesis-, and lipolysis-related gene in eWAT of HFD mice at mRNA levels (n = 6). *M* and *N*, Western blot analysis of PPARγ and FABP4 at protein levels, and the result of β-actin is the same as Figure 3*A* (n = 6). Data are represented as means ± SEM. Significance was determined using *t* tests. ∗*p* < 0.05, ∗∗*p* < 0.01. eWAT, epididymal white adipose tissue; GTT, glucose tolerance test; HFD, high-fat diet; ITT, insulin tolerance test; iWAT, inguinal white adipose tissue; WAT, white adipose tissue.

**Figure S1:**
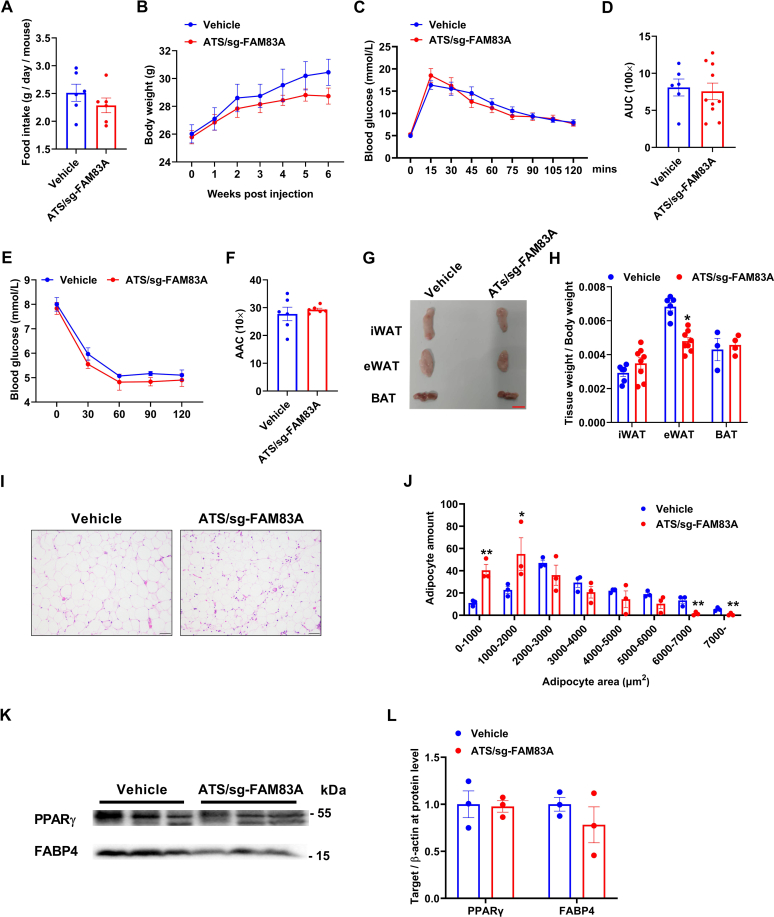
**The effects of adipose-specific KO of *Fam83a* on system metabolism in mice fed with NCD.***A*, analysis of food intake in the experiment (n = 6). *B*, changes of body weight (n = 6). *C* and *D*, GTT assay (n = 6). *E* and *F*, ITT assay (n = 6). *G* and *H*, morphological comparison (*G*) and weight (*H*) of iWAT, eWAT, and BAT (n = 6). The scale bar represents 1 cm. *I* and *J*, H&E staining of the eWAT section (n = 6). The scale bar represents 100 μm. *K* and *L*, Western blot analysis of PPARγ and FABP4 at protein levels in the eWAT of NCD mice, and the result of β-actin is the same as Figure 2*E* (n = 6). Data are represented as means ± SEM. Significance was determined using *t* tests. ∗*p* < 0.05, ∗∗*p* < 0.01. eWAT, epididymal white adipose tissue; GTT, glucose tolerance test; HFD, high-fat diet; ITT, insulin tolerance test; iWAT, inguinal white adipose tissue; NCD, normal chow diet.

**Figure S2:**
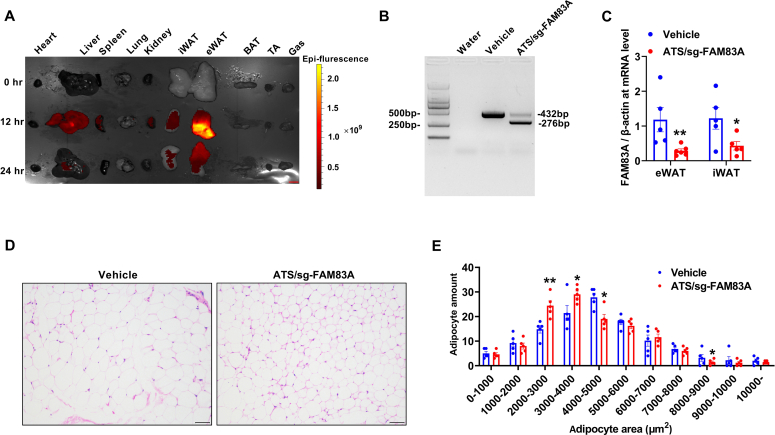
**Supportive information about adipose-specific KO of *Fam83a* in mice fed with HFD.***A*, tracing FITC fluorescence in various tissues of mice. The scale bar represents 1 cm. *B*, agarose gel electrophoresis image about the KO efficiency of ATS//sg-FAM83A in eWAT of HFD mice at genomic level. *C*, the mRNA expression of *Fam83a* in eWAT and iWAT of HFD mice (n = 6). *D* and *E*, H&E staining of iWAT in HFD mice. The scale bar represents 100 μm. Data are represented as means ± SEM. Significance was determined using *t* tests. ∗*p* < 0.05, ∗∗*p* < 0.01. eWAT, epididymal white adipose tissue; HFD, high-fat diet; iWAT, inguinal white adipose tissue.

Male C57BL/N mice were fed with normal chow diet (NCD) for 6 weeks (from 8 weeks to 16 weeks old), and there was no significant difference in the food intake or body weight observed between ATS/sg-FAM83A mice and the vehicle control (Fig. S1, *A* and *B*). Glucose tolerance tests (GTTs) and insulin tolerance tests (ITTs) also presented similar results between the 2 groups (Fig. S1, *C–F*). Although, tissue weights (Fig. S1, *G* and *H*) and adipocyte size (Fig. S1, *I* and *J*) of eWAT seemed significantly reduced upon *Fam83a* inhibition. Expression levels of adipogenic, lipogenic, or lipolytic marker genes/protein were evenly matched between ATS/sg-FAM83A and control mice, except for a significant decrease of *Fabp4* mRNA (Fig. S1, *K* and *L*). High-fat diet (HFD) always leads to dramatic fat expansion, thus another counterpart of ATS/sg-FAM83A mice were fed with HFD for 6 weeks (∼40g in body weight). As expected, body weights were dramatically increased upon the stimuli of HFD and knockdown of *Fam83a via* injection of complex of FITC-ATS-9R and FAM83A-sgRNA/Cas9 (Fig. 3*A*), although food intake were not significantly different (Fig. 3*B*). In HFD condition, FITC-ATS-9R oligoplexes fluorescence were mainly enriched in both eWAT and iWAT (Fig. S2*A*). To compare the data in NCD mice, eWAT was focused in the following assay. The knockdown efficiency of *Fam83a* at genomic (Fig. S2*B*), mRNA (Fig. S2*C*), and protein levels (Fig. 3*C*) were confirmed by PCR, reverse transcription quantitative PCR (RT-qPCR), and Western-blot, respectively. Tissue weights of iWAT and eWAT were significantly reduced after *Fam83a* knockdown (Fig. 3*E*), and the adipocyte areas of the eWAT (Fig. 3, *F* and *G*) and iWAT (Fig. S2, *D* and *E*) in ATS/sg-FAM83 A mice became significantly smaller than those in the control group. GTT (Fig. 3, *H* and *I*) and ITT (Fig. 3, *J* and *K*) results showed that glucose tolerance and insulin-mediated glucose clearance in ATS/sg-FAM83A-injected mice had been significantly improved. Consistent with the smaller adipocytes, mRNAs levels of adipogenic (*Pparγ*, *Fabp4*, *C/ebpα*, *Adipoq*), lipogenic (*Srebp1c*, *Acsl1*, *Fasn*, *Dgat1*, *Dgat2*), and lipolytic (*Pnpla2*, *Lipe*, *Lpl*) marker genes were significantly downregulated in eWAT of ATS/sg-FAM83A mice (Fig. 3*L*). In line with that, protein levels of PPARγ and FABP4 were also reduced (Fig. 3, *M* and *N*). These results indicate that loss of *Fam83a* protects mice from diet-induced obesity.

### KO/knockdown *Fam83a* inhibits adipocyte differentiation *in vitro*

**Figure 4:**
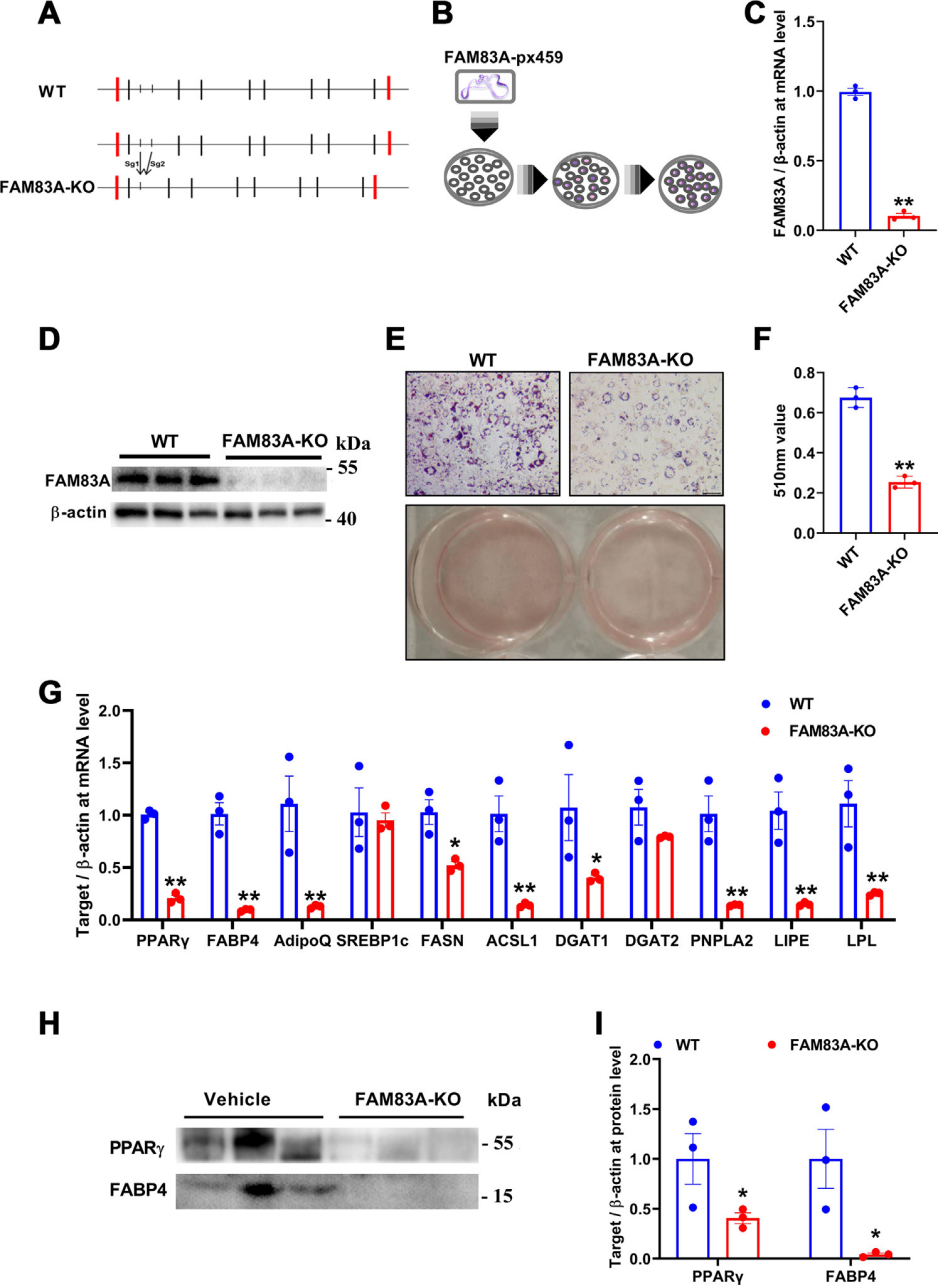
**Knockout of FAM83A with CRISPR/Cas9 inhibits adipogenic differentiation in 3T3-L1 cells.***A*, diagram of FAM83A KO strategy. *B*, diagram to construct FAM83A-KO 3T3-L1 cell line. *C*, detection of *Fam83a* mRNA expression with RT-qPCR (n = 3). *D*, detection of FAM83A protein expression by Western blot (n = 3). *E*, *oil red* O staining. The scale bar represents 100 μm. *F*, statistic assay of *oil red* O staining. *G*, changes of adipogenesis-, lipogenesis-, and lipolysis-related genes at mRNA level (n = 3). *H* and *I*, Western blot analysis of PPARγ and FABP4 at protein levels, and the result of β-actin is the same as Figure 4*D* (n = 3). Data are represented as means ± SEM. Significance was determined using *t* tests. ∗*p* < 0.05, ∗∗*p* < 0.01. RT-qPCR, reverse transcription quantitative PCR.

To further explore the role of *Fam83a* during adipogenesis *in vitro*, FMA83A-KO cell line was constructed using CRISPR-Cas9 with specific guide RNAs that targeted the first exon of *Fam83a* genome (Fig. 4*A*). After transfection of the pX459 plasmid, positive clones were selected by puromycin (Fig. 4*B*). The KO efficiency of *Fam83a* in FMA83A-KO cell line was further confirmed by the detection of *Fam83a* expression at mRNA (Fig. 4*C*) and protein levels (Fig. 4*D*). Six days after adipogenic differentiation, significantly fewer lipid droplets (indicated by oil red O staining) were detected in FAM83A-KO compared with those in WT cell line (Fig. 4, *E* and *F*). Consistently, expression of adipogenic (*Pparγ*, *Fabp4*, *Adipoq*), lipogenic (*Srebp1c*, *Fasn*, *Acsl1*, *Dgat1*, *Dgat2*), and lipolytic (*Pnpla2*, *Lipe*, *Lpl*) genes were significantly downregulated in the FAM83A-KO cell line (Fig. 4*G*), and protein expression of adipogenic marker genes, PPARγ and FABP4, were also reduced after *Fam83a* KO (Fig. 4, *H* and *I*).

**Figure S3:**
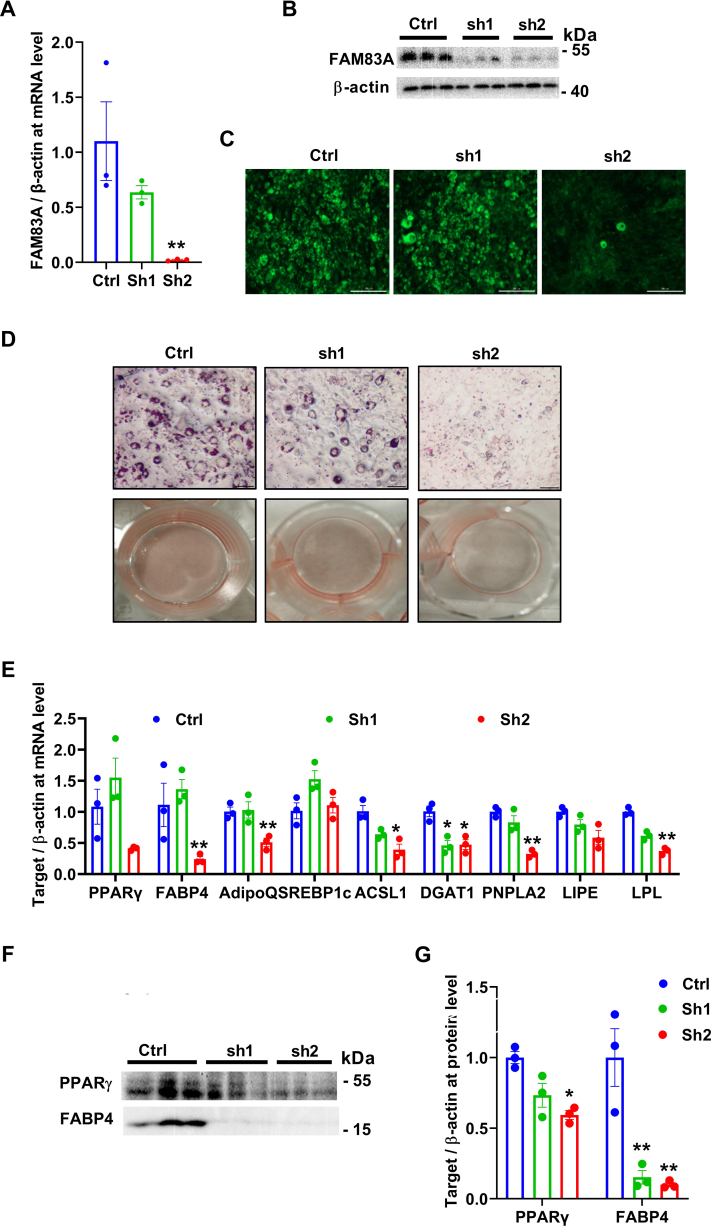
**Knockdown of *Fam83a* with lentivirus-shRNA hampered adipogenesis in 3T3-L1 cells.***A*, detection of knockdown efficiency using RT-qPCR (n = 3). *B*, detection of knockdown efficiency using Western blot (n = 3). *C*, Bodipy staining. *D*, oil red O staining. The scale bar represents 100 μm. *E*, expression of adipogenesis-, lipogenesis-, and lipolysis-related gene at mRNA levels (n = 3). *F* and *G*, expression of PPARγ and FABP4 at protein levels, and the result of β-actin is the same as Figure S3*B* (n = 3). Data are represented as means ± SEM. Significance was determined using *t* tests. ∗*p* < 0.05, ∗∗*p* < 0.01. RT-qPCR, reverse transcription quantitative PCR.

To further confirm the role of FAM83A in adipocytes *in vitro*, 3T3-L1 cell line that stably expressing lentivirus-*Fam83a* shRNA was generated. The knockdown efficiency of lentivirus-*Fam83a* shRNA on *Fam83a* expression was confirmed by real-time PCR and sh2-FAM83A resulted in a better knockdown (Fig. S3*A*). The interference efficiency of sh2-FAM83A was further validated by Western blot (Fig. S3*B*). Oil red O staining showed that lipid droplets formation was also inhibited in sh-FAM83Acells after 6 days of differentiation (Fig. S3, *C* and *D*). In addition, expression levels of adipogenic (*Pparγ*, *Fabp4*, *Adipoq*), lipogenic (*Srebp1c*, *Acsl1*, *Dgat1*), and lipolytic (*Pnpla2*, *Lipe*, *Lpl*) marker genes in sh2-FAM83A were significantly decreased (Fig. S3*E*), and significant reduction was detected in PPARγ and FABP4 expression at protein levels (Fig. S3, *F* and *G*). These results suggest that KO and knockdown of *Fam83a* inhibits adipogenic differentiation of 3T3-L1 cells *in vitro*.

### Inhibition of *Fam83a* leads to mitochondrial damage

**Figure 5:**
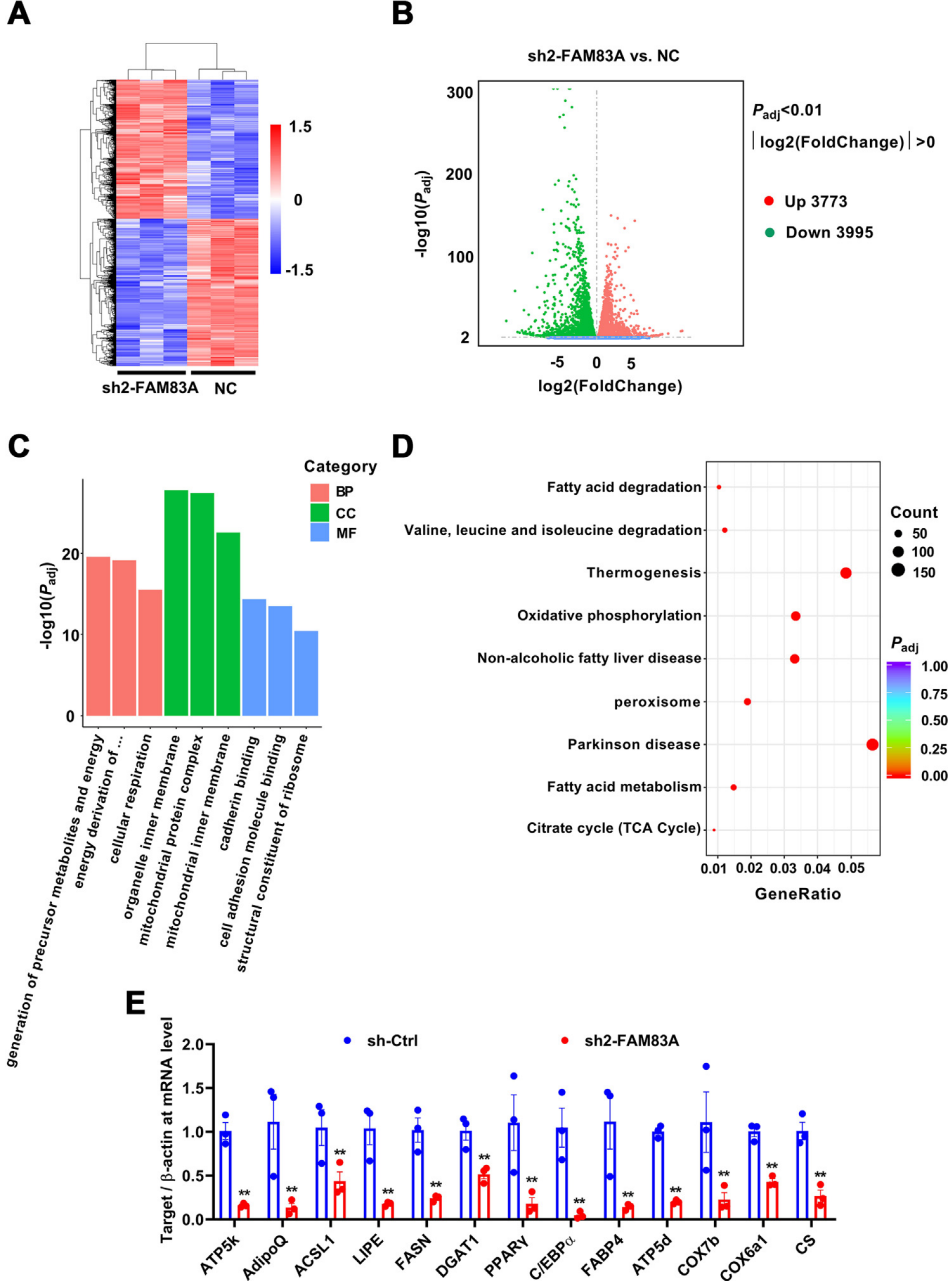
**RNA-seq assay on 3T3-L1 cells transfected with shRNA-FAM83A or negative control.***A*, heatmap representing the global gene expression between sh-Ctrl and sh2-FAM83A. *B*, volcano plot representing differential expressed gene between sh-Ctrl and sh2-FAM83A 3T3-L1 cell lines. *C*, GO assay. (*D*, KEGG pathway analysis (n = 3). *E*, changes of differentially expressed genes related with mitochondrial function were verified with RT-qPCR. GO, gene ontology; RT-qPCR, reverse transcription quantitative PCR.

To explore the underlying mechanisms how FAM83A regulates adipocyte differentiation, total RNA was isolated from well-differentiated sh-Ctrl and sh2-FAM83A 3T3-L1 cells for RNA-seq (Fig. 5*A*). Among the differential expression genes (DEGs), 3773 were upregulated and 3995 were downregulated DEGs by knockdown of *Fam83a* (Fig. 5*B*). Gene ontology analysis revealed that lots of DEGs were specifically related to mitochondria functions (Fig. 5*C*). The results of the Kyoto Encyclopedia of Genes and Genomes (KEGG) analysis also showed that downregulated DEGs were mainly enriched in the lipid metabolism of adipocytes and tricarboxylic acid cycle (Fig. 5*D*). The expression patterns of DEGs that associated with adipogenic differentiation and mitochondrial aerobic respiration in RNA-seq were further verified by RT-qPCR and lipid metabolism–associated (*Adipoq*, *Acsl1*, *Lipe*, *Fasn*, *Dgat1*, *Pparγ*, *C*/*ebpα*, *Fabp4*) and mitochondria-associated (*Atp5k*, *Atp5d*, *Cox7b*, *Cox6a1*, *Cs*) genes were all downregulated after *Fam83a* knockdown (Fig. 5*E*).

**Figure 6:**
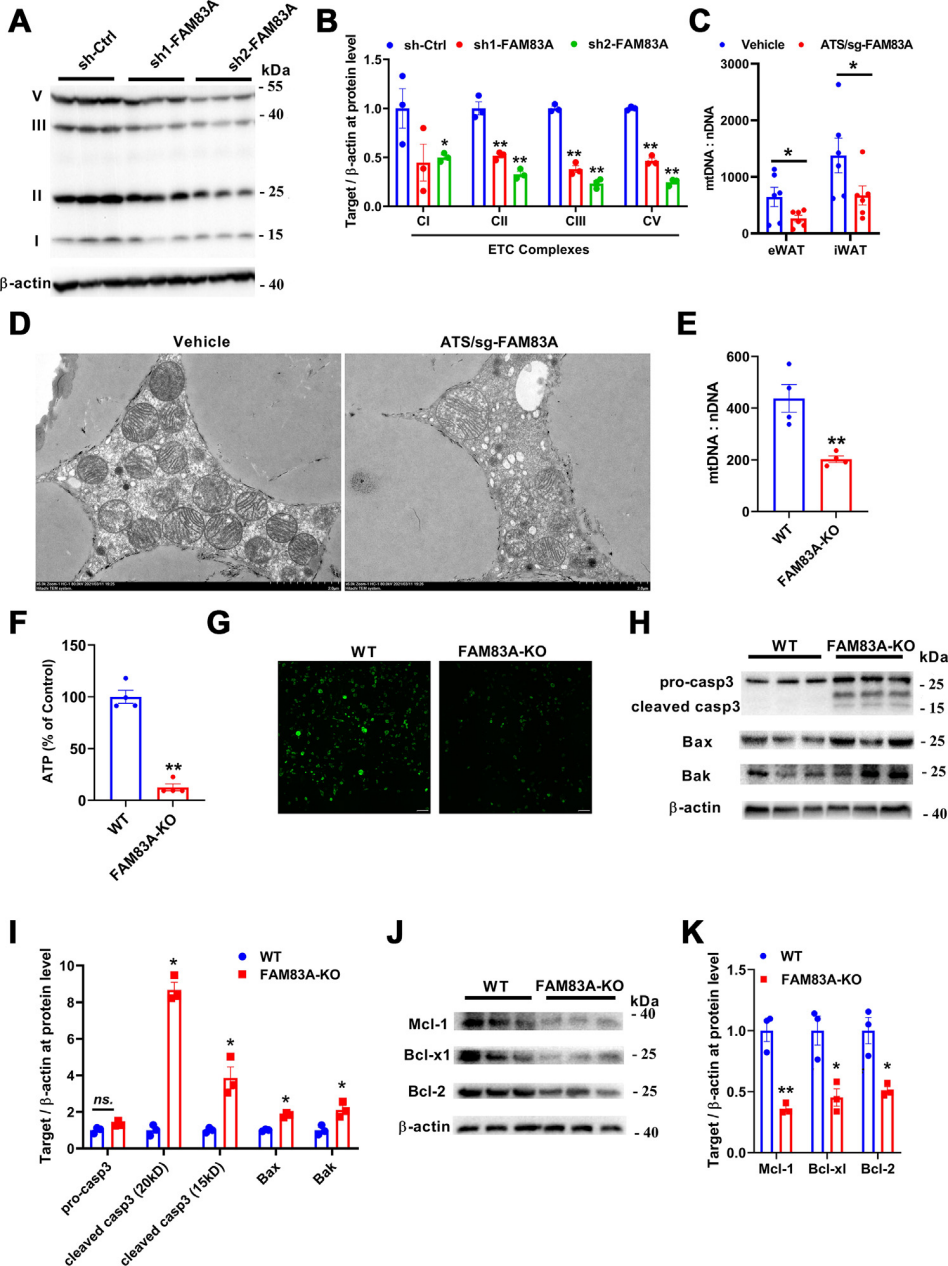
**Repression of FAM83A in adipocytes results in mitochondrial damage.***A* and *B*, Western blot analysis of ETC complex at protein level in differentiated 3T3-L1 cells transfected with shRNAs. *C*, analysis of mitochondria copy number in eWAT of HFD mice (n = 6). *D*, electron microscope images of mitochondria in eWAT of HFD mice. The scale bar represents 2 μm. *E*, analysis of mitochondria copy number in differentiated 3T3-L1 adipocytes with or without KO of *Fam83a* (n = 3). *F*, ATP production in differentiated 3T3-L1 adipocytes with or without KO of *Fam83a* (n = 3). *G*, MPTP assay in differentiated 3T3-L1 adipocytes with or without KO of *Fam83a* (n = 3). The scale bar represents 100 μm. *H* and *I*, Western blot analysis of apoptosis-related factors at protein levels (n = 3). *J* and *K*, Western blot analysis of antiapoptosis factors at protein levels (n = 3). Data are represented as means ± SEM. Significance was determined using *t* tests. ∗*p* < 0.05, ∗∗*p* < 0.01. eWAT, epididymal white adipose tissue; HFD, high-fat diet; MPTP, mitochondrial permeability transition pore.

Specially, Western blot showed that proteins of that from 4 complexes of the mitochondrial electron transport chain were largely reduced in sh2-*Fam83a* cells (Fig. 6, *A* and *B*). In ATS/sg-FAM83A mice fed with HFD described previously, mitochondrial copy number in eWAT and iWAT was significantly reduced (Fig. 6*C*). Furthermore, transmission electron microscope analysis was applied to detect the ultrastructure of mitochondria, and results indicated that mitochondria were swollen and the copy number of mitochondria was decreased in the electron microscope section of the eWAT of HFD mice (Fig. 6*D*). Moreover, in *Fam83a* KO cell line generated by CRISPR-Csa9 (6 days post adipogenic induction), mitochondrial copy number and ATP production were also decreased (Fig. 6, *E* and *F*), and mitochondrial permeability transition pore (MPTP) test also indicated that loss of *Fam83a* disrupted the permeability of mitochondrial inner membrane (Fig. 6*G*). It is well known that mitochondria damage always leads to cell apoptosis (25, 26). Consistently, apoptotic markers, such as cleaved caspase-3, BCL2 associated X (Bax), and Bak, were significantly increased after *Fam83a* KO (Fig. 6, *H* and *I*), while apoptotic inhibitors, Mcl-1, Bcl-x1, and Bcl-2 were significantly decreased in *Fam83a* KO cells (Fig. 6, *J* and *K*). These lines of evidence together indicate that *Fam83a* deficiency could lead to mitochondrial damage and induce apoptosis in white adipocytes.

### Mitochondria damage leads to metabolic defect in ATS/sg-FAM83A mice after HFD

**Figure 7:**
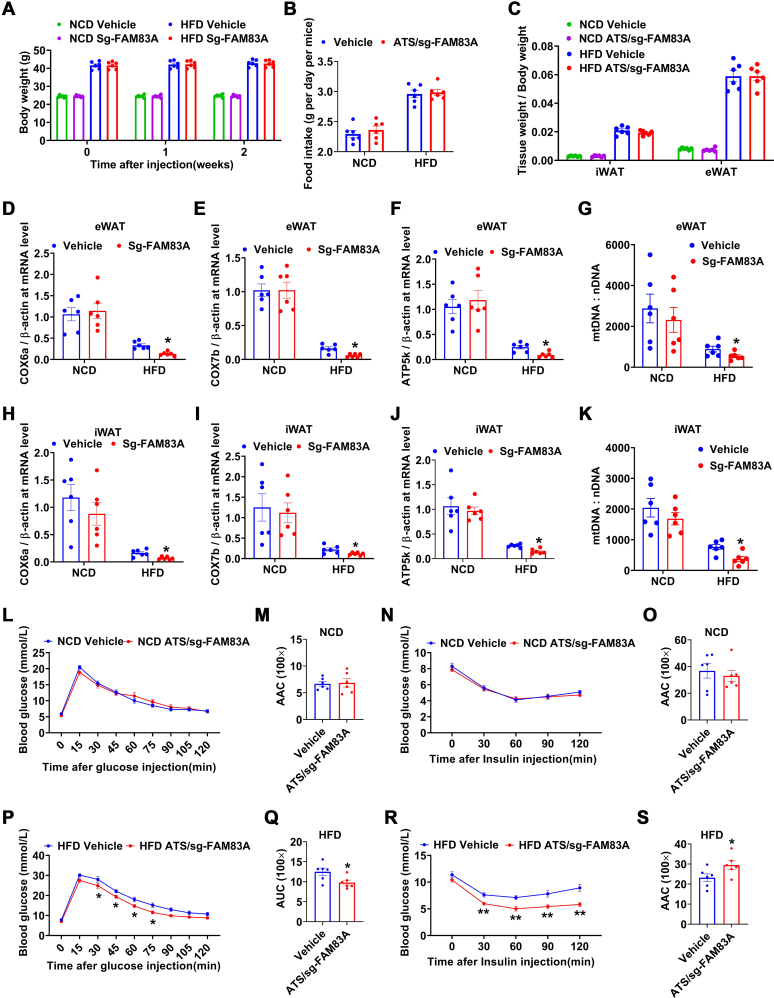
**Damage to adipose mitochondria prior to HFD-induced metabolic alteration.***A*–*C*, body weight (*A*), food intake (*B*), and tissue index (*C*) of mice fed with NCD or HFD (n = 6). *D*–*G*, the mRNA expression of *COX6a1* (*D*), *COX7b* (*E*), *ATP5K* (*F*), and mitochondrial copy number (*G*) in the eWAT of HFD mice (n = 6). *H*–*K*, the mRNA expression of *COX6a1* (*H*), *COX7*b (*I*), *ATP5K* (*J*), and mitochondrial copy number (*K*) in the eWAT of mice (n = 6). *L*–*S*, GTT and ITT on NCD or HFD mice. The AUC was determined for each individual animal for GTT, and the AAC was determined for each individual animal for ITT. Data are represented as means ± SEM. Significance was determined using *t* tests. ∗*p* < 0.05, ∗∗*p* < 0.01. eWAT, epididymal white adipose tissue; GTT, glucose tolerance test; HFD, high-fat diet; ITT, insulin tolerance test; NCD, normal chow diet.

Mitochondria damage in adipocytes could directly alter insulin sensitivity in mice (9, 27). Thus we moved on to test whether reduced WAT mass were resulted from the mitochondria damage induced by *Fam83a* inhibition. Male WT and ATS/sg-FAM83A mice of 8-week-old were fed with either NCD or HFD for 2 weeks; no significant difference was observed in either body weight (Fig. 7*A*), food intake (Fig. 7*B*), or adipose mass (Fig. 7*C*) in NCD or HFD mice. However, mitochondrial function related genes, *cox6a*, *cox7b*, *ATP5k*, as well as mitochondrial copy number in eWAT (Fig. 7, *D*–*G*) and iWAT (Fig. 7, *H*–*K*), were significantly reduced in ATS/sg-FAM83A HFD but not ATS/sg-FAM83A NCD mice. Notably, GTT and ITT in ATS/sg-FAM83A HFD instead of ATS/sg-FAM83A NCD mice (Fig. 7, *L*–*S*). Taken together, these experiments indicated that mitochondrial damage is the primary consequence of *Fam83a* inhibition that leads to the AT mass loss and improved insulin sensitivity.

### Downregulation of FAM83A leads to mitochondrial damage by CK1

**Figure 8:**
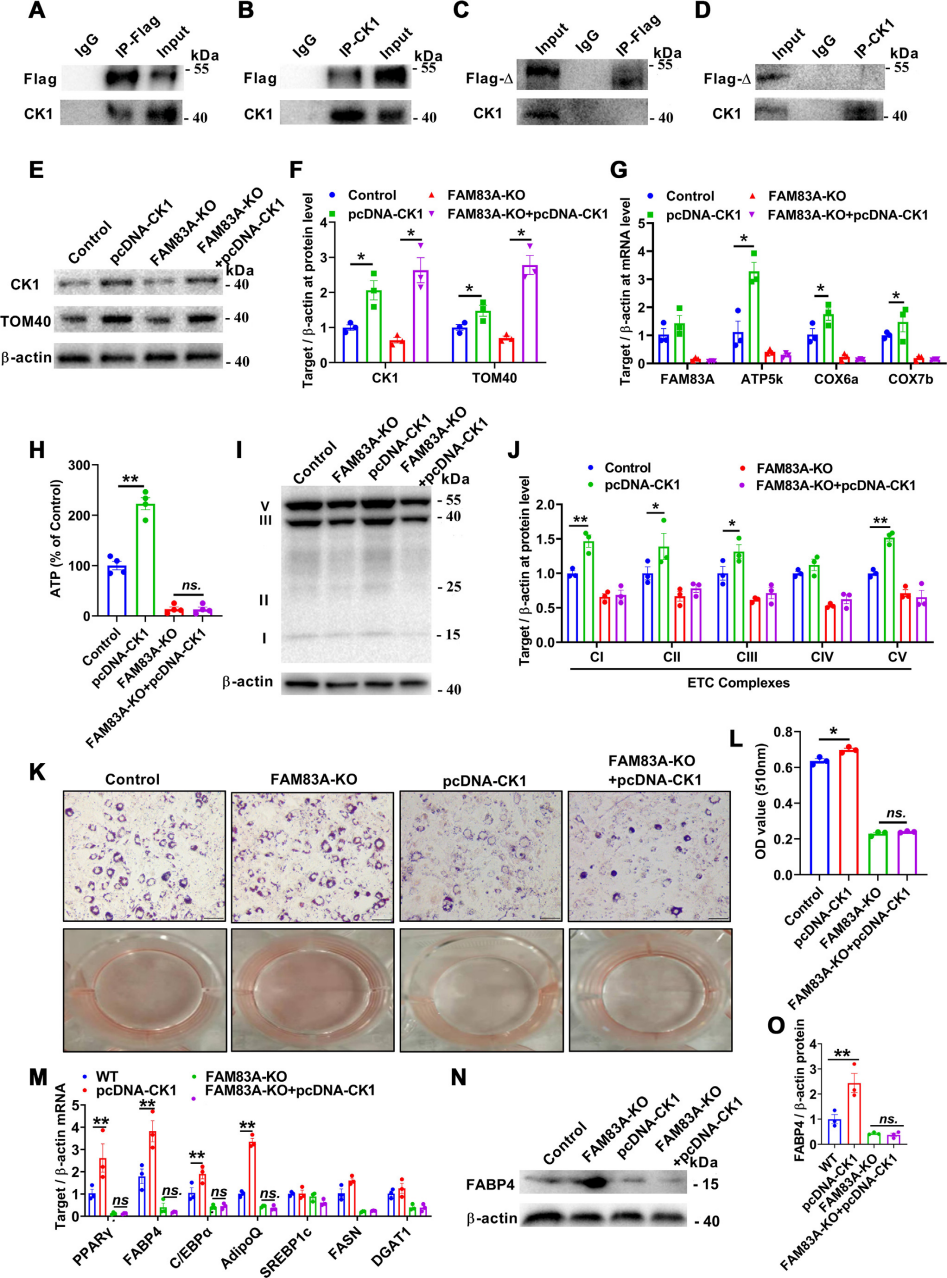
**KO of *Fam83a* impairs CK1-modulated mitochondrial function.***A* and *B*, coIP assay revealed an interaction between FAM83A and CK1 using anti-flag (*A*) or anti-CK1 (*B*). *C* and *D*, coIP assay revealed no interaction between FAM83A-Δ1669 mutants and CK1. *E* and *F*, detection of CK1 and TOM40 expression at protein levels with Western blot. *G*, analysis of *Fam83a*, *ATP5K*, *COX6a1*, and *COX7b* at mRNA levels using RT-qPCR. *H*, comparison of ATP production. (*I*, *J*, Western blot analysis of ETC complex at protein levels. *K* and *L*, oil red O staining (*K*) and statistical analysis (*L*). The scale bar represents 100 μm. *M*, analysis of lipogenesis-related genes at mRNA levels using RT-qPCR (n = 3). *N* and *O*, Western blot analysis of FABP4 at protein levels (n = 3). Data are represented as means ± SEM. Significance was determined using *t* tests. ∗*p* < 0.05, ∗∗*p* < 0.01. coIP, coimmunoprecipitation; RT-qPCR, reverse transcription quantitative PCR.

**Figure S4:**
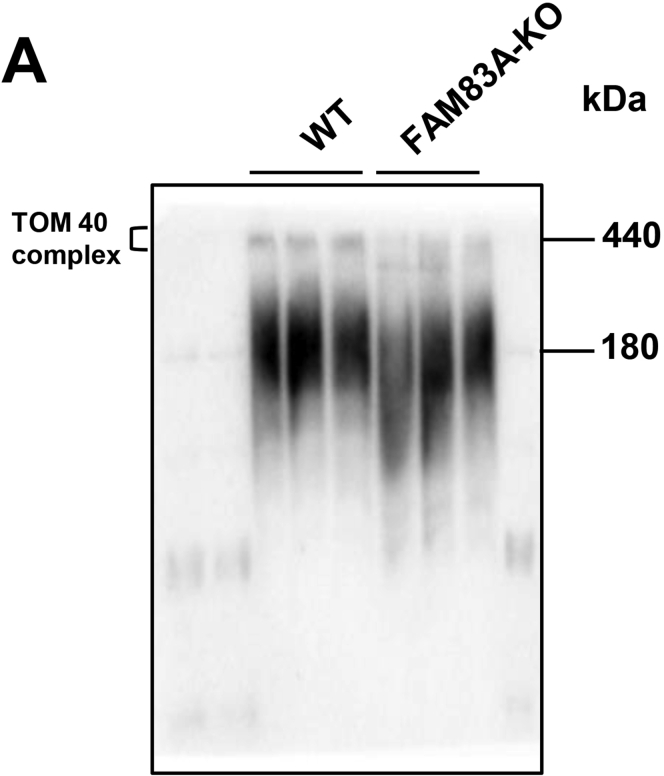
**KO of *Fam83a* with CRISPR/Cas9 inhibits formation of the TOM40 complex in 3T3-L1 cells.***A* native-PAGE analysis of TOM40 complex at protein level in 3T3-L1 cells (n = 3).

FAM83A has been reported to interact with CK1 in yeast cells, which is an important protein involved mitochondrial outer membrane protein transport (28, 29). Coimmunoprecipitation assay showed that FAM83A could directly interact with CK1 in 3T3-L1 cell line (Fig. 8, *A* and *B*), and deletion of the well-known interaction domain, DUP1669 domain of *Fam83a*, dramatically eliminated the interaction between FAM83A and CK1 (Fig. 8, *C* and *D*). The protein level of TOM40 complex, which serves as a downstream target of CK1 and play key roles in maintaining the permeability of the outer mitochondrial membrane, was significantly decreased after *Fam83a* knockdown (Fig. S4*A*). To further confirm whether the FAM83A modulates mitochondria function in a CK1-dependent manner, pcDNA-*Ck1* was constructed, and transfection of pcDNA-*Ck1* into 3T3-L1 cells significantly enhanced the expression of TOM40 in WT cells but failed in *Fam83a*-KO 3T3-L1 cell lines (Fig. 8, *E* and *F*). In line with that, overexpression of *Ck1* promoted the expression levels of mitochondria-related genes (Fig. 8*G*) and ATP production (Fig. 8*H*) in WT but not *Fam83a*-KO 3T3-L1 cells. Protein levels in the mitochondrial electron transport chain presented a similar result (Fig. 7, *I* and *J*). It has been reported that CK1 is a positive regulator of adipose differentiation (16, 17). Similarly, overexpression of *Ck1* promoted adipogenic differentiation in WT but not *Fam83a*-KO 3T3-L1 cells (Fig. 8, *K* and *L*). Moreover, the mRNA levels of the adipogenic and lipogenic marker genes and the protein levels of FABP4 also showed similar changes (Fig. 8, *M*–*O*). Therefore, CK1 was required in the modulatory effects of FAM83A on mitochondria function and adipogenic differentiation.

## Discussion

*Fam83a* is previously described as an oncogenic gene, while its function in metabolic organs, especially ATs, remains largely unclear. In the current study, we invested a novel role of Fam83a during adipogenesis of white adipocytes. Loss of function of *Fam83a* in WAT inhibited fat accumulation and resisted diet-induced obesity while improving insulin sensitivity. Knockdown or KO of *Fam83a* in the 3T3-L1 cell line disrupted adipocyte differentiation *in vivo*. Particularly, inhibition of *Fam83a* decreased mitochondria number and ATP production both *in vivo* and *in vitro*. In addition, repression of *Fam83a* also induced apoptosis in adipocytes. Mechanistic studies indicated that FAM83A interacted with CK1 and impaired adipogenesis caused by *Fam83a* knockdown was due to CK1-mediated mitochondrial maintenance (Fig. 8). Thus, FAM83A is potentially offered as a novel target for the treatment of obesity and type II diabetes.

There are only scattered reports about the function of FAM83A in adipose tissues previously. An early research demonstrated that FAM83A promotes the proliferation of the 3T3-L1 cell line (20) and KO of *Zfp217*, which dramatically inhibits adipocyte differentiation, results in a significant increase of *Fam83a* expression (22). Our data also showed that FAM83A protein was relatively highly expressed in WAT and differentiated adipocytes, indicating that *Fam83a* may be a positive regulator of adipogenic differentiation. With the help of powerful deliver system of FITC-ATS-9R (24, 30), adipose-specific KO of *Fam83a* in mice (ATS/sg-FAM83A mice) were generated. Results showed that ATS/sg-FAM83A mice could alleviate HFD-induced obesity and type II diabetes. Moreover, inhibition of *Fam83a* expression with CRISPR/Cas9 or shRNA-lentivirus also resulted in significant reduction of lipid accumulation and adipogenic genes expression in 3T3-L1 cells *in vitro*. Therefore, our work verified FAM83A as a novel and positive modulator on adipogenic differentiation.

Intriguingly, KO of *Fam83a* in adipose pads ameliorated glucose tolerance and insulin-induced glucose clearance in mice upon HFD stimuli, and meanwhile, suppressed FAM83A function resulted in serious mitochondrial damage in adipocytes both *in vivo* and *in vitro*. Accumulated documents have shown that mitochondria are important for adipogenic differentiation, and damage to mitochondria in adipocytes inhibited adipogenesis (9, 31). Given that mitochondrial damage and increased insulin sensitivity in mice were also reported previously (9, 27), we moved on to explore the potential relationship between mitochondrial damage and metabolic alteration in ATS/sg-FAM83A mice. Data revealed that, in ATS/sg-FAM83A HFD mice, mitochondrial damage could be detected before metabolism status was changed, indicating that the damaged mitochondria resulted from loss of function of *Fam83a* might hamper adipose expansion, and the latter might contribute to system metabolism.

Research has shown that the DUF1669 domain of the FAM83 family binds mechanically to different isoforms of CK1 *in vitro* (32). Our results showed that FAM83A binds to CK1 in adipocytes, which was consistent with the hypothesis that the FAM83 family regulates the cellular localization of various subtypes of CK1 (32). CK1 promotes the phosphorylation of TOM22 to facilitate the assembly of the TOM40 complex and maintain the smoothness of the mitochondrial outer membrane protein transport channel (33, 34). Moreover, CK1 is a positive regulator of adipogenic differentiation (16, 17). Thus, the CK1-mediated regulation of mitochondria plays an important role in adipocyte differentiation. In parallel, adipocytes apoptosis is also consistent with the abnormal mitochondrial outer membrane function (35, 36). Our data also showed that knockdown of FAM83A leads to increased levels of apoptosis in adipocytes which may be directly responsible for weight loss in ATS/sg-FAM83A HFD mice.

**Figure 9:**
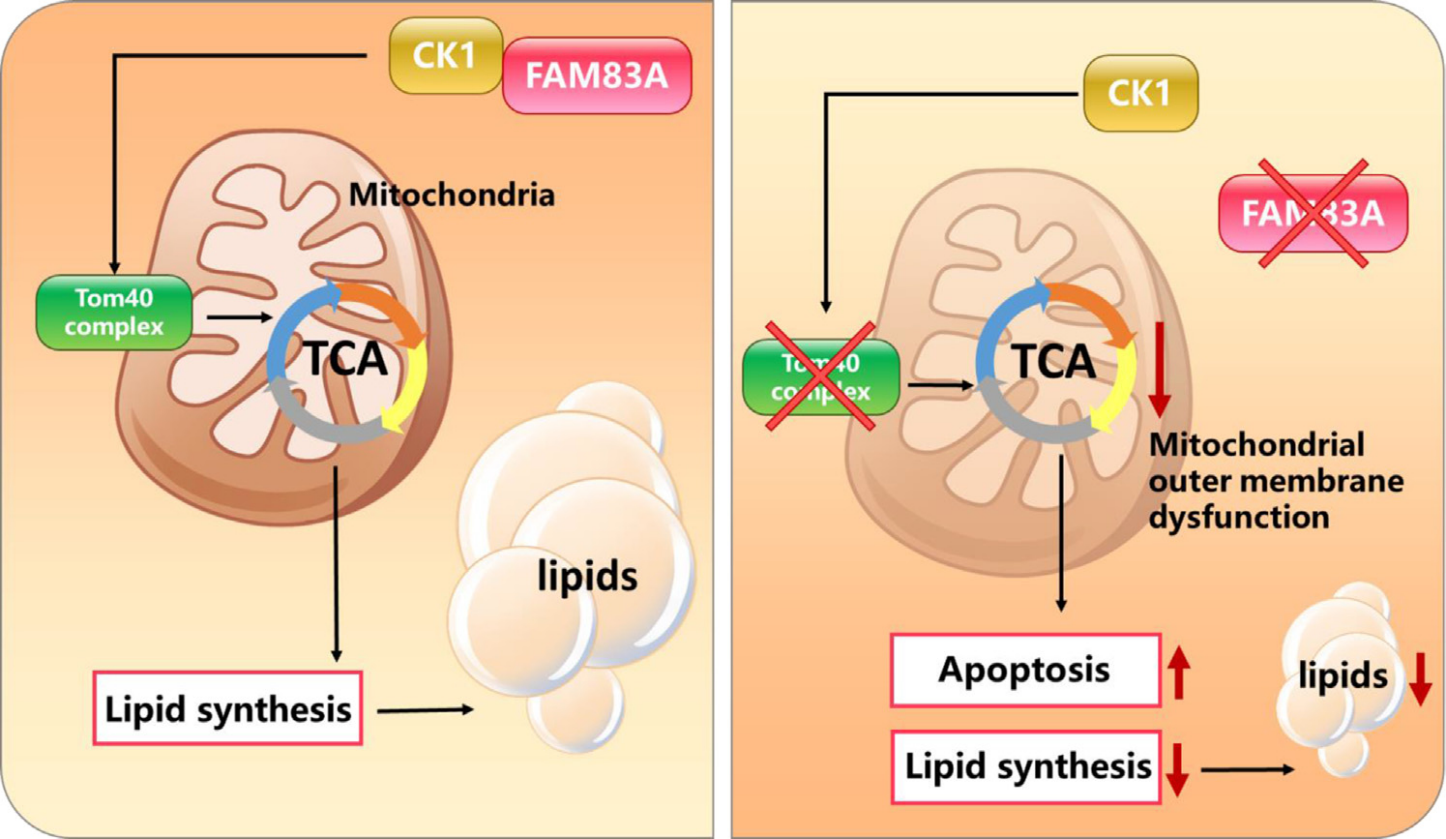
**Schematic diagram of FAM83A affecting adipogenesis.** In the presence of FAM83A, FAM83A interacted with CK1, and then CK1 promoted the formation of the TOM40 complex to maintain the permeability of the mitochondrial membrane and ensure the normal function of mitochondria in adipocytes, which was necessary for adipogenic differentiation in adipocytes (left). When FAM83A is lacking, CK1 could not bind to FAM83A or be localized on the mitochondria, which resulted in the failure to assemble the TOM40 complex. Lacking in the TOM40 complex leads to abnormal mitochondrial membrane permeability, which causes mitochondrial damage and induces apoptosis, and ultimately inhibits adipocyte differentiation (right).

In conclusion, we found that knockdown of *Fam83a* inhibits diet-induced obesity and type II diabetes *in vivo* and suppresses adipocyte differentiation *in vitro*. Mechanistic studies show that knockdown of *Fam83a* inhibits mitochondrial aerobic respiration by blocking the CK1 pathway, maintaining the permeability of the outer mitochondrial membrane (Fig. 9). *Fam83a* gene offers a novel potential target for the treatment of obesity and type II diabetes.

## Experimental procedures

### Animals

ATS/sg-FAM83A mice were produced using male C57BL-6J mice, which were purchased from the Animal Center of Xi'an Jiaotong University. ATS/sg-FAM83A NCD mice were injected with FITC-ATS-9R peptide and 2 FAM83A-sgRNA/Cas9 plasmids, which were incubated half an hour before i.p. injection, and injection was done twice weekly at a dose of 0.35 mg/kg for 6 weeks. The vehicle NCD mice were injected with FITC-ATS-9R peptide and empty-pX459 plasmids that were incubated half an hour in advance twice weekly at a dose of 0.35 mg/kg for 6 weeks. ATS/sg-FAM83A or vehicle HFD mice were fed high fat from high fat until they reached 40 g body weight for oligoplex injection in the same way as in normal mice. Mice were fed HFD (TP23400; Nantong Trophy Feed Technology Co, Ltd), which consisted of 60% fat or NCD (D12450B; JiangSu Xietong Biological Co, Ltd). The body weight of mice was monitored weekly. Mice were housed on a 12 h light/dark cycle and provided ad libitum access to food and water. All animal protocols were approved by the Committee of Experimental Animal Management at Northwest Agriculture and Forestry University, China.

### Cell culture

The 3T3-L1 cell lines were cultured in growth medium Dulbecco's Modified Eagle Medium (DMEM) with 10% fetal bovine serum (FBS) at 37 °C in a humidified atmosphere with 5% carbon dioxide. When cells reached 100% confluence, they were cultured with DMEM and 10% FBS and supplemented with 10 μg/ml insulin, 1 μM dexamethasone, and 0.5 mM isobutyl methylxanthine (Sigma–Aldrich) for 2 days. Then, the medium was changed to DMEM with 10% FBS and 5 μg/ml insulin for a further 4 days to maintain differentiation.

### FAM83A subcellular localization

The FAM83A-N-EGFP plasmid vector was constructed by General Biological Co, Ltd using pEGFP-C1 plasmid. The FAM83A-N-EGFP plasmid was transfected in the 3T3-L1 cell line. After 48 h, MitoTracker Red CMXRos (YESEN Biotechnology Co, Ltd, 40741ES50) staining was performed for 30 min and fixed in 4% paraformaldehyde for 10 min. Cells were then 4′,6-diamidino-2-phenylindole stained for 10 min and photographed with Revolution WD Confocal.

### Construction of the FAM83A-sgRNA/Cas9 plasmid vector

FAM83A-sgRNA/Cas9 plasmid vector was constructed using pX459 plasmid, which was purchased from Addgene (#48139). Two candidate sequences to target *Mus musculus* FAM83A were designed on the Zhangfeng Lab guide-design website (https://zlab.bio/guide-design-resources).

FAM83A-sgRNA1/Cas9 F: CACCGGGCAAGATCCGGAAACGTC

FAM83A-sgRNA1/Cas9 R: AAACGACGTTTCCGGATCTTGCCC

FAM83A-sgRNA2/Cas9 F: CACCGGTAGACTTCCTGTCCTCAG

FAM83A-sgRNA2/Cas9 R: AAACCTGAGGACAGGAAGTCTACC

The primer sequences of genotyping for the FAM83A-KO.

FAM83A-sgRNA/Cas9-g F: CTACGTCTGGAAGAGCTCCG

FAM83A-sgRNA/Cas9-g R: TCAGCCAAAGTCCAGGTGTG

### Construction of the FAM83A-KO cell line

FAM83A-sgRNA1/Cas9 and FAM83A-sgRNA2/Cas9 plasmids were transfected in the 3T3 cell line, and positive cells were selected with puromycin (3 ug/ml) for 48 h. Next, a single clone of the positive cell was picked in a 96-well plate, and genotyping was performed after they had grown to a certain number. Finally, the cell line with only positive clones was selected as the FAM83A-KO cell line.

### Synthesis and characterization of FAM83A-sgRNA/Cas9 + FITC-ATS-9R oligoplexes

The peptides FITC-ATS-9R (CKGGRAKD-RRRRRRRRRC) were purchased from YaoQiang Biotechnology Co, Ltd. The molecular weights of FITC-ATS-9R were 2844 Da. To prepare each sample, 10 μg of FAM83A-sgRNA1/Cas9 and FAM83A-sgRNA2/Cas9 were condensed with FITC-ATS-9R in normal saline for 30 min. Electrophoretic mobility shift assays were performed to confirm the FITC-ATS-9R condensation properties. Oligoplexes with different amounts of FITC-ATS-9R were prepared with a constant amount of FAM83A-sgRNA/Cas9 in normal saline. After 30 min of incubation, samples were electrophoresed on 0.8% agarose gels (Lonza) in Tris-borate-EDTA buffer solution at 120 V for 30 min and observed using an imaging station.

### Lentivirus packaging and construction of the sh-FAM83A lentiviral cell line

Two candidate sequences to target *M. musculus* FAM83A (NM_173862.2) were designed on the Thermo Fisher Scientific BLOCK-iT RNAi Designer website (https://zlab.bio/guide-design-resources). The pLKO.1-Puro plasmid was gifted by the Kuang Laboratory of Purdue University. The 293T cell line was transfected with shRNA:pSPAX2:pMD2.G at a ratio of 4:3:1. The culture media was changed after 8 to 12 h, and the media supernatant was harvested at 48 h. The collected supernatant was filtered with a 0.45 μm filter to become the packaged lentivirus. The 3T3-L1 cell line was cultivated, the packaged lentivirus was added to the media, and the media were then changed after 8 to 12 h. The 3T3-L1 cell line was selected with puromycin for more than 72 h.

sh1-FAM83A

Forward: CCGGGTGTGGAAGGGGAGGTGTACTCGAGTACACCTCCCCTTCCACACTTTTTG

Reverse: AATTCAAAAAGTGTGGAAGGGGAGGTGTACTCGAGTACACCTCCCCTTCCACAC

sh2-FAM83A

Forward: CCGGGGACTGGACTCCTGCTCTTTACTCGAGTAAAGAGCAGGAGTCCAGTCCTTTTTG

Reverse: AATTCAAAAAGGACTGGACTCCTGCTCTTTACTCGAGTAAAGAGCAGGAGTCCAGTCC

### WAT histology analysis

Adipose tissue was isolated and fixed in 4% paraformaldehyde and maintained at 4 °C until use. The fixed tissues were dehydrated and processed for paraffin embedding, and 5 μm sections were stained with H&E. Adipocyte size was determined using ImageJ (US National Institutes of Health) and measured a minimum of 300 cells per group. For oil red O staining, we used 10 μm sections.

### Metabolic studies

For the GTT, mice were fasted overnight (12–16 h), and an i.p. injection of glucose (0.75 g/kg body weight) was administered. For the ITT, mice were fasted for 4 h before receiving an i.p. injection of insulin (1.5 U/kg body weight). Blood glucose concentrations were measured at 0, 15, 30, 45, 60, 75, 90, 105, and 120 min after glucose injection and 0, 30, 60, 90, and 120 min after insulin injection.

### RT-qPCR

Total RNA was extracted from the differentiated 3T3-L1 cell line or AT using the TRIzol reagent (Invitrogen). Using the PrimeScript RT Reagent Kit (Takara Biomedical Technology), 1 μg of RNA was reverse transcribed into complementary DNA. PCR amplification was performed using the SYBR PCR mix (Takara Biomedical Technology Co). The primers for quantitataive PCR are listed in Table S1.

### Western blot analysis

Briefly, 15 μg of total lysates from tissues or cells were run on a 10% SDS-PAGE and immunoblotted with the primary antibodies (1:1000) for FAM83A (Wuhan Dai'an Biotechnology Co, Ltd, C1640), PPARγ (Abcam, ab3442), FABP4 (Santa Cruz, sc-271529), caspase-3 (Santa Cruz, sc-1225), BAX (Santa Cruz, sc-20067), β-actin (ProteinTech, 66009-1-Ig), oxidative phosphorylation (OXPHOS) (Abcam, ab110413), Bcl-2 (ABWAYS, CY5032), Mcl1 (ABWAYS, CY5199), Bak (ABWAYS, CY5372), Bcl-x1 (ABWAYS, CY5050), caspase 3 (Wanleibio, WL02117), TOM40 (Santa Cruz, sc-365467), and CK1 (Santa Cruz, sc-74582). The intensity of bands was measured using ImageJ or Imagelab. All experiments were repeated at least 3 times and mean values were derived.

### RNA-seq analysis

Total RNA was extracted from sh-Ctrl and sh2-FAM83A 3T3-L1 cell lines. The RNA-seq experiments were performed by Novogene company. The transcriptome library for sequencing was generated using a KAPA-stranded RNA-seq Library Prep Kit (Illumina) following manufacturer recommendations. The clustering of the index-coded samples used the KAPA RNA Adapters set1/set2 for Illumina. After clustering, the libraries were sequenced on the Illumina HiSeq X Ten platform using a (2 × 150 bp) paired-end module. The DEGs were identified by a *p*-value < 0.05 and a fold-change of >1.5 between the 2 groups.

### Bodipy staining of lipid droplets

Cells were washed with PBS 3 times and fixed with 4% paraformaldehyde for 10 min at room temperature (RT). Subsequently, cells were incubated with BODIPY (Invitrogen) for 30 min and washed 3 times with PBS. Finally, images were captured using a fluorescence microscope (Nikon).

### Oil red O staining and dye extraction analysis

The 3T3-L1 cell lines were fixed with 4% paraformaldehyde for 10 min at RT. Subsequently, the fixed cells were washed and stained with 1% filtered oil red O for 30 min, washed, and observed using phase-contrast microscopy (IS-Elements software, Nikon Eclipse).

### Mitochondrial DNA quantitation

Quantification of relative copy number differences was carried out using analysis of the difference in threshold amplification between mitochondrial DNA and nuclear DNA (delta-delta Ct method) (37). The mitochondrial DNA and nuclear DNA primers are included in Table S1.

### ATP assay

Cells were lysed and centrifuged at 12,000*g* for 5 min at 4 °C, and the supernatants were collected. Standard and ATP working solutions were prepared according to ATP assay kit instructions. The ATP assay working solution was added to the samples and standards for measurement. ATP assay was conducted using the ATP assay kit according to kit instructions (Beyotime, S0026).

### MPTP assay

The cells were washed twice with PBS, and each well was incubated with calcein-AM staining solution, fluorescence quenching solution, or t Ionomycin control for 30 min away from light. The media were then replaced with fresh media and incubated for 30 min and protected from light. The cells were then washed in PBS 3 times before fluorescent photography. MPTP assay was conducted using the MPTP Assay Kit according to kit instructions (Beyotime, C2009s)

### Coimmunoprecipitation assay

3T3-L1 cells were transfected with 3xFLAG-CMV-10 plasmid (containing WT *Fam83a* or mutant *Fam83a* lacking DUP1669 domain) and harvested 48 h later to extract total protein. The lysate was precleared with protein A/G dynabeads at 4 °C for 1 h. Then 2 mg of primary antibody (1:500) anti-flag (ProteinTech, 66008-3-Ig) and anti-CK1 (ProteinTech, 55192-1-AP) were added to the lysate containing 500 mg total protein and rotated at 4 ^°^C overnight. The following morning, protein A/G agarose was added, and the lysate was rotated for 2 h. The samples were washed with cold PBS 3 times and collected for Western blot analysis.

### Statistical analysis

All replicate experiments (including cell- and mouse-based experiments) were biological replicates that were repeated at least 3 times. All analyses were performed using SPSS version 23 (SPSS Inc). All data are represented as means ± SEM. Comparisons between the 2 groups were made using *t* tests. Statistical significance was represented as follows: ∗*p* < 0.05, ∗∗*p* < 0.01.

## Data availability

The data that support the findings of this study are available from the corresponding author upon reasonable request.

## Supporting information

Supporting Information includes one table and four figures.

## Conflict of interest

The authors declare that they have no conflicts of interest with the contents of this article.
