# Supplementary material for: Proto-oncogene FAM83A contributes to casein kinase 1–mediated mitochondrial maintenance and white adipocyte differentiation
Source: J Biol Chem. 2022 Aug 2;298(10):102339. doi: 10.1016/j.jbc.2022.102339 (PMC9493395; doi:10.1016/j.jbc.2022.102339)
Supplement: Supplemental Table S1 [file mmc1.docx]

Table S1. Primer sequences used in RT-qPCR

| Gene | Primer sequence | Length | Tm/°C |
| --- | --- | --- | --- |
| PPARγ | F: TCGCTGATGCACTGCCTATG  R: GAGAGGTCCACAGAGCTGATT | 103 | 60 |
| FABP4 | F: AAGGTGAAGAGCATCATAACCCT  R: TCACGCCTTTCATAACACATTCC | 133 | 60 |
| ADIPOQ | F: TGTTCCTCTTAATCCTGCCCA  R: CCAACCTGCACAAGTTCCCTT | 104 | 60 |
| SREBP1c | F: GATGTGCGAACTGGACACAG  R: CATAGGGGGCGTCAAACAG | 104 | 60 |
| FASN | F: GGAGGTGGTGATAGCCGGTAT  R: TGGGTAATCCATAGAGCCCAG | 140 | 60 |
| ACSL1 | F: TGCCAGAGCTGATTGACATTC  R: GGCATACCAGAAGGTGGTGAG | 101 | 60 |
| DGAT1 | F: TCCGTCCAGGGTGGTAGTG  R: TGAACAAAGAATCTTGCAGACGA | 199 | 60 |
| DGAT2 | F: GCGCTACTTCCGAGACTACTT  R: GGGCCTTATGCCAGGAAACT | 172 | 60 |
| PNPLA2 | F: CAACGCCACTCACATCTACGG  R: GGACACCTCAATAATGTTGGCAC | 106 | 62 |
| LIPE | F: CCAGCCTGAGGGCTTACTG  R: CTCCATTGACTGTGACATCTCG | 106 | 60 |
| LPL | F: GGGAGTTTGGCTCCAGAGTTT  R: TGTGTCTTCAGGGGTCCTTAG | 115 | 60 |
| C/EBPα | F: CAAGAACAGCAACGAGTACCG  R: GTCACTGGTCAACTCCAGCAC | 124 | 60 |
| β-actin | F: GTCCCTGACCCTCCCAAAAG  R: GCTGCCTCAACACCTCAACCC | 266 | 60 |
| ATP5k | F: GTTCAGGTCTCTCCACTCATCA  R: CGGGGTTTTAGGTAACTGTAGC | 95 | 60 |
| ATP5d | F: TGCTTCAGGCGCGTACATAC  R: CACTTGCTTGACGTTGGCA | 128 | 60 |
| COX7b | F: TTGCCCTTAGCCAAAAACGC  R: TCATGGAAACTAGGTGCCCTC | 101 | 60 |
| COX6a1 | F: TCAACGTGTTCCTCAAGTCGC  R: AGGGTATGGTTACCGTCTCCC | 115 | 60 |
| CS | F: GGACAATTTTCCAACCAATCTGC  R: TCGGTTCATTCCCTCTGCATA | 109 | 60 |
| FAM83A | F: AGTGATAATGAGAGCGCCCG  R: CGAAGCCACAGGGAAGTAGG | 243 | 60 |
